# Deficiency in the omega-3 lysolipid transporter Mfsd2a leads to aberrant oligodendrocyte lineage development and hypomyelination

**DOI:** 10.1172/JCI164118

**Published:** 2023-06-15

**Authors:** Vetrivel Sengottuvel, Monalisa Hota, Jeongah Oh, Dwight L. Galam, Bernice H. Wong, Markus R. Wenk, Sujoy Ghosh, Federico Torta, David L. Silver

**Affiliations:** 1 Signature Research Program in Cardiovascular and Metabolic Disorders and; 2 Centre for Computational Biology, Duke-NUS Medical School, Singapore, Singapore.; 3 Singapore Lipidomics Incubator, Life Sciences Institute, and; 4 Precision Medicine Translational Research Programme and Department of Biochemistry, Yong Loo Lin School of Medicine, National University of Singapore, Singapore, Singapore.

**Keywords:** Metabolism, Neuroscience, Demyelinating disorders, Monogenic diseases, Neurodevelopment

## Abstract

Patients with autosomal recessive microcephaly 15 caused by deficiency in the sodium-dependent lysophosphatidylcholine (LPC) transporter major facilitator superfamily domain–containing 2a (Mfsd2a) present with both microcephaly and hypomyelination, suggesting an important role for LPC uptake by oligodendrocytes in the process of myelination. Here we demonstrate that Mfsd2a is specifically expressed in oligodendrocyte precursor cells (OPCs) and is critical for oligodendrocyte development. Single-cell sequencing of the oligodendrocyte lineage revealed that OPCs from OPC-specific Mfsd2a-KO mice (2aOKO mice) underwent precocious differentiation into immature oligodendrocytes and impaired maturation into myelinating oligodendrocytes, correlating with postnatal brain hypomyelination. 2aOKO mice did not exhibit microcephaly, a finding consistent with the notion that microcephaly is the consequence of an absence of LPC uptake at the blood-brain barrier rather than a deficiency in OPCs. Lipidomic analysis showed that OPCs and iOLs from 2aOKO mice had significantly decreased levels of phospholipids containing omega-3 fatty acids, with a corresponding increase in unsaturated fatty acids, the latter being products of de novo synthesis governed by Srebp-1. RNA-Seq indicated activation of the Srebp-1 pathway and defective expression of regulators of oligodendrocyte development. Taken together, these findings indicate that the transport of LPCs by Mfsd2a in OPCs is important for maintaining OPC state to regulate postnatal brain myelination.

## Graphical abstract

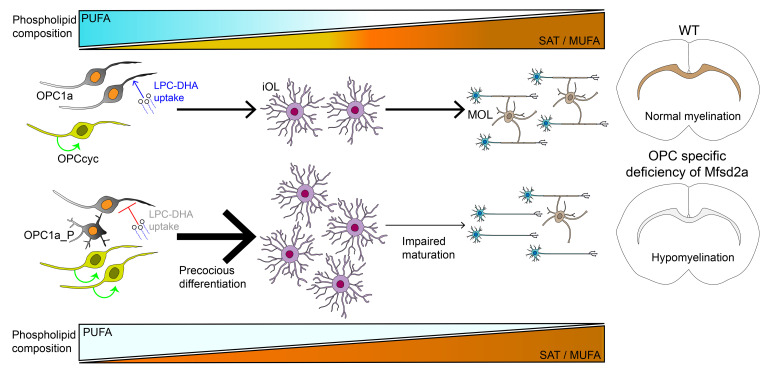

## Introduction

Myelin is a conserved membrane structural component of axons derived from the plasma membrane of oligodendrocytes that is essential for saltatory axon conduction and axon energetics (1–3). Myelination in the central nervous system orchestrated by myelinating oligodendrocytes (MOLs) is a dynamic process, the majority of which occurs postnatally, continues throughout life, and is modified by neural activity and environment (4–9). Recent single-cell sequencing studies have determined that formation of MOLs is a linear developmental process beginning with oligodendrocyte precursor cells (OPCs) derived from neural progenitors and progresses in the following order: differentiation into committed oligodendrocyte progenitors (COPs), newly formed oligodendrocytes (NFOLs), myelin-forming oligodendrocytes (MFOLs), and finally MOLs (10, 11).

De novo lipogenesis is driven by SREBPs, transcription factors that are important for the regulation of genes that maintain cellular lipid homeostasis (12). Srebp proteins form a complex with its chaperone protein sterol cleavage-activating protein (Scap) that is essential for the regulated proteolytic activation of Srebp (13). Genetic deficiency of Scap in mature oligodendrocytes (MFOL and MOL) in mice resulted in delayed myelination, but persistent hypomyelination if Scap was dually deleted from both astrocytes and mature oligodendrocytes (14). These findings indicated that de novo synthesis of cholesterol is essential in myelination of MOLs, and astrocytes might be playing a supportive role in myelination by supplying cholesterol to oligodendrocytes. It is not known whether oligodendrocytes take up other types of exogenous lipids, such as phospholipids, that could act as extrinsic factors to regulate maintenance of OPC pools, oligodendrocyte differentiation, and myelination. Moreover, in which oligodendrocyte lineage stage lipid transport might be important for myelination is also presently unclear.

Lipidomic analysis of mouse and human myelin indicates that 75% of the myelin membrane contains glycerophosholipids (e.g., phosphatidylcholine [PC], phosphatidylethanolamine [PE], phosphatidylserine [PS]), with minor amounts of sphingolipids (8%) and cholesterol (20%) (15, 16). The most abundant fatty acid species within the phospholipids of myelin are saturated and monounsaturated fatty acids (16). Biosynthesis of a massive amount of membrane phospholipids is believed to be exclusively derived from de novo biosynthesis within cells of the brain, and acquisition of essential fatty acids such as docosahexaenoic acid (DHA) from the periphery into the developing brain via the lipid transporter protein major facilitator superfamily domain containing 2a (Mfsd2a) (17). Mfsd2a is a sodium-dependent lysophosphatidylcholine (LPC) transporter highly expressed by the endothelium of the blood-brain barrier (BBB) and the blood-retinal barrier (BRB), as well as other tissues, such as liver, lung, kidney, and placenta (18–20), and is the major pathway for brain and eye accretion of DHA (17, 21). Mfsd2a does not transport unesterified DHA, but rather transports DHA esterified as LPC (LPC-DHA), with a substrate preference for LPCs containing mono- and polyunsaturated fatty acids. Recently solved cryo–electron microscopy (cryo-EM) structures of Mfsd2a suggest that a flippase-type mechanism underlies transport (22). Mice with gene-targeted deletion of Mfsd2a (2aKO mice) exhibited severe microcephaly and brain DHA deficiency (17, 23). Importantly, humans with loss-of-function mutations in MFSD2A (also known as microcephaly 15 autosomal recessive) presented with severe microcephaly and hypomyelination (24–27), indicating that LPC transport is critical for brain growth and myelination. We recently provided genetic and biochemical evidence that Mfsd2a is required at the BBB during postnatal life to mediate normal brain growth linked to neuron arborization (28). Mechanistically, LPC-DHA treatment of primary neural stem cells that express Mfsd2a resulted in downregulation of Srebp-1 processing and activation in an Mfsd2a-dependent fashion, resulting in profound effects on phospholipid membrane saturation (28). These data identify LPC-DHA transported by Mfsd2a as an extrinsic physiological regulator of membrane phospholipid composition, acting in part through regulation of Srebp-1 activity during postnatal brain development. While these studies have provided a biochemical mechanism by which LPC-DHA functions in postnatal brain growth, they have not explained a mechanism for the prominent hypomyelination phenotype observed in humans with Mfsd2a loss-of-function mutations.

In the current study, we provide evidence that Mfsd2a is expressed in the oligodendrocyte lineage, specifically in oligodendrocyte precursors, and that Mfsd2a plays an important role in maintaining the precursor cell state through regulation of phospholipid pools containing polyunsaturated fatty acids that negatively affect oligodendrocyte maturation and myelination. These studies reveal that LPCs act as extrinsic factors to regulate OPC state.

## Results

### Loss of Mfsd2a in OPCs causes hypomyelination.

Patients with Mfsd2a-inactivating mutations exhibit severe hypomyelination in addition to microcephaly (24–27). Consistent with these characteristics, mice with gene-targeted deletion of Mfsd2a (2aKO mice) displayed hypomyelination and microcephaly (Supplemental Figure 1A; supplemental material available online with this article; https://doi.org/10.1172/JCI164118DS1). Transcriptomic data from the literature indicated that in addition to endothelial cells of the BBB and astrocytes, Mfsd2a is expressed in the oligodendrocyte lineage, with expression restricted to OPCs, COPs, and NFOLs (10, 11, 29). To confirm the expression of Mfsd2a in OPCs, we utilized our Mfsd2a lineage tracing line crossed to a Rosa26: CAG-tdtomato reporter line (2aKI-ERT2-cre: tdtomato) (30). Tamoxifen induction of the 2aKI-ERT2-cre: tdtomato transgene in mouse pups at P10 showed colocalization with Pdgfra^+^ cells in the mouse brain parenchyma, confirming Mfsd2a expression in OPCs (Supplemental Figure 1B).

**Figure 1:**
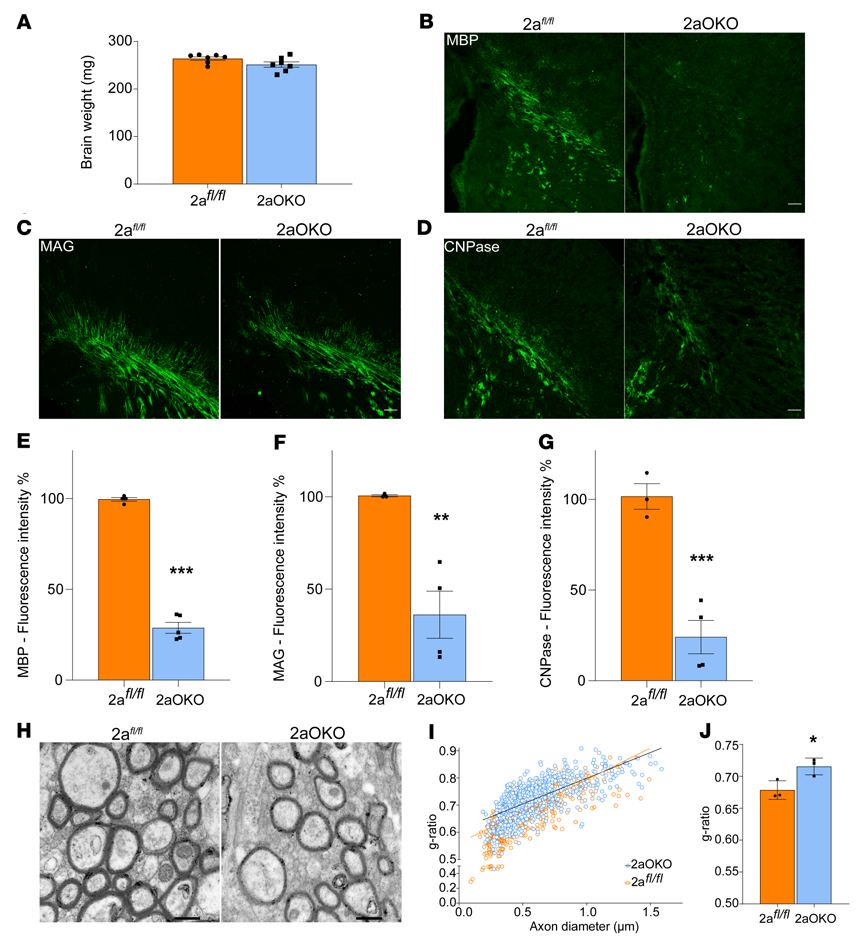
Loss of Mfsd2a in OPCs causes hypomyelination. (**A**) P8 brain weights of 2a*^fl/fl^* and 2aOKO mouse pups indicate no microcephaly due to Mfsd2a knockout in OPCs. Data are presented as mean ± SEM; *n* = 7 per genotype. (**B**–**G**) Representative images and quantification of coronal sections of the corpus callosum from P8 brain immunostained with the myelin marker proteins MBP (**B** and **E**), MAG (**C** and **F**), and CNPase (**D** and **G**) indicate hypomyelination in 2aOKO compared with 2a*^fl/fl^* control mice. Data are presented as mean ± SEM; *n* = 3–5 per genotype. Scale bars: 100 μm. ***P < 0.0001, **P < 0.001 by 2-tailed Student’s *t* test (unpaired). (**H**) Representative TEM images of the corpus callosum of P67 brains from 2a*^fl/fl^* and 2aOKO mice. Scale bars: 500 nm. (**I**) Graphical representation of the g-ratio of individual fibers in relation to axon diameter presented as scatter plots for 2a*^fl/fl^* and 2aOKO mice. (**J**) Histogram of g-ratio comparison between 2a*^fl/fl^* and 2aOKO mice. Data are presented as mean ± SEM; *n* = 3 per genotype. *P < 0.05 by 2-tailed Student’s *t* test (unpaired).

To determine whether genetic deletion of Mfsd2a in OPCs would result in defects in myelination, we generated an OPC-specific Mfsd2a deletion mouse model using a floxed allele of Mfsd2a (2a*^fl/fl^*) crossed to the OPC cre driver line Pdgfra (referred to herein as 2aOKO mice; see Table 1 for a key to mouse lines used in this study). 2aOKO mice had normal brain weights at P8, which differed from the severe microcephaly exhibited by mice with conventional or endothelium-specific knockout of Mfsd2a (Figure 1A) (28). Immunofluorescence (IF) on P8 brain sections using major myelin marker proteins — myelin basic protein (MBP), myelin-associated glycoprotein (MAG), and 2′,3′-cyclic nucleotide-3′-phosphodiesterase (CNPase) — showed that levels of all 3 myelin proteins were significantly reduced in 2aOKO relative to control mice (Figure 1, B–G). To independently confirm these findings, we generated 2 other knockout mouse models for Mfsd2a in OPCs: one line using a Sox10-cre driver and another using the tamoxifen-inducible Plp1-ERT-cre driver. Importantly, in mice with deletion of Mfsd2a in OPCs using these cre driver lines, myelination was also greatly reduced — without an effect on brain weight — relative to that in control brains (Supplemental Figure 1, C and D). Transmission electron microscopy (TEM) on P67 corpus callosum to analyze maximal myelination in adult brain showed that myelin thickness on axons as determined by g-ratio was significantly reduced in 2aOKO mice relative to 2a*^fl/fl^* controls (Figure 1, H–J). These findings demonstrate that loss of Mfsd2a in OPCs led to hypomyelination during early postnatal development.

### Mfsd2a deficiency in OPCs alters the population dynamics of the oligodendrocyte lineage.

To delineate whether the early postnatal hypomyelination observed in 2aOKO mice was due to changes in oligodendrocyte lineage differentiation, we used Ribo-TRAP mouse lines specific for OPC (Pdgfra-EGFP/Rpl10a) and the whole oligodendrocyte lineage (Olig2-EGFP/Rpl10a) that allow for isolation of distinct subtypes of cells for single-cell RNA-Seq as well as cell phenotype characterization (31, 32). Crossing 2a*^fl/fl^* or 2aOKO mice with the OPC-specific Ribo-TRAP line generated 2a*^fl/fl^*-OPC-Ribo-TRAP control and 2aOKO-OPC-Ribo-TRAP lines; crossing 2a*^fl/fl^* or 2aOKO mice with the whole oligodendrocyte lineage–specific Ribo-TRAP line generated 2a*^fl/fl^*-OL-Ribo-TRAP control and 2aOKO-OL-Ribo-TRAP lines. Given the reduced postnatal developmental myelination seen in 2aOKO mice, we focused our analysis on P8, a time point at which OPCs proliferate and undergo differentiation, and one that has been previously and extensively characterized by single-cell sequencing (10, 29), thus providing a transcriptional roadmap for our studies. FACS sorting of GFP^+^ cells from 2a*^fl/fl^*-OPC-Ribo-TRAP mouse brain showed that cell isolation could be performed at approximately 95% purity, as determined by GFP and Olig2 coimmunostaining of sorted cells (Supplemental Figure 2A). IF analysis showed that the majority of GFP^+^ cells in 2a*^fl/fl^*-OPC-Ribo-TRAP brains were Pdgfra^+^ and in 2a*^fl/fl^*-OL-Ribo-TRAP brains were Olig2^+^, confirming the specificity of these reporter lines for detecting OPCs and the entire oligodendrocyte lineage population, respectively (Supplemental Figure 2, B and C).

**Figure 2:**
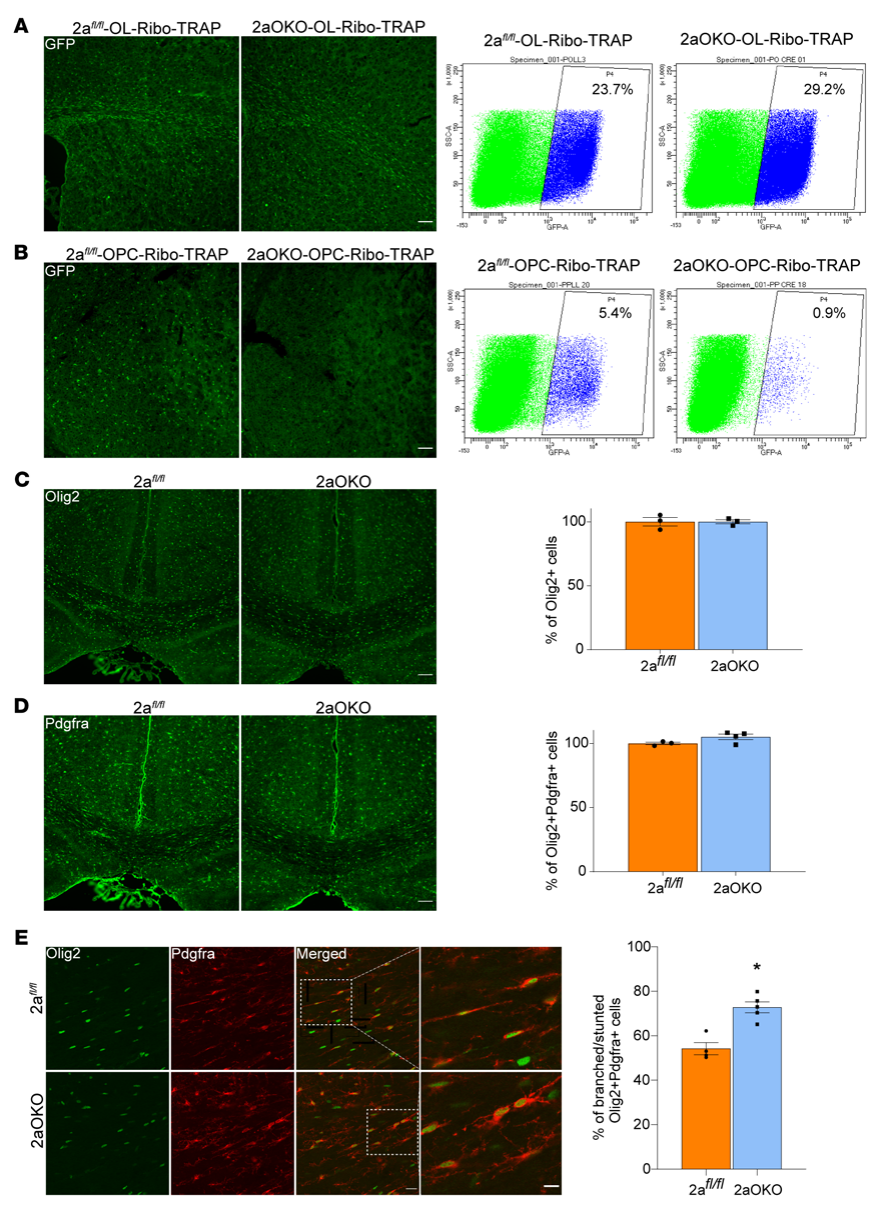
Mfsd2a deficiency in OPCs alters their numbers and morphology. (**A**) Flow cytometry analysis of GFP^+^ cells from P8 brains of 2a*^fl/fl^*-OL-Ribo-TRAP control and 2aOKO-OL-Ribo-TRAP mice indicates no major changes in the total oligodendrocyte lineage population resulting from Mfsd2a deficiency in OPCs. *n* = 3 per genotype. (**B**) Flow cytometry analysis of GFP^+^ cells from P8 2a*^fl/fl^*-OPC-Ribo-TRAP control and 2aOKO-OPC-Ribo-TRAP brains revealed a decrease in GFP^+^ OPCs in 2aOKO-OPC-Ribo-TRAP brains. *n* = 3 per genotype. (**C**) IF analysis of P8 brain coronal sections from 2a*^fl/fl^* and 2aOKO mice with Olig2 antibody indicates similar numbers of whole oligodendrocyte lineage cells at the corpus callosum in these genotypes. *n* = 3 per genotype. (**D**) Double immunostaining of P8 brain coronal sections with Pdgfra and Olig2 antibodies indicates similar Pdgfra^+^ OPC population numbers at the corpus callosum in 2a*^fl/fl^* and 2aOKO mice. *n* = 3 per genotype. Scale bars: 100 μm. (**E**) Double immunostaining from **D** reveals OPC morphological heterogeneity, with an approximately 25% increase in cells with branched/stunted morphology in 2aOKO compared with 2a*^fl/fl^* control mice, which mostly display a classical bipolar morphology. Scale bar: 20 μm. The far-right columns show 2.5× magnified images of the regions indicated in the merged images. Scale bar: 10 μm. Data are presented as mean ± SEM; *n* = 3–4 per genotype.*P < 0.01 by 2-tailed Student’s *t* test (unpaired).

FACS sorting of GFP^+^ cells from 2a*^fl/fl^*-OL-Ribo-TRAP and 2aOKO-OL-Ribo-TRAP lines showed no significant difference in the total Olig2^+^ cell population (23.7% and 29.2% respectively) (Figure 2A). However, FACS sorting of GFP^+^ cells from the 2a*^fl/fl^*-OPC-Ribo-TRAP and 2aOKO-OPC-Ribo-TRAP lines showed a remarkable 80% reduction in the Pdgfra^+^ cell population (5.4% and 0.9%, respectively) (Figure 2B). IF analysis performed using an Olig2 antibody on 2a*^fl/fl^* and 2aOKO mouse brains showed no difference in the total numbers of whole oligodendrocyte lineage cells, consistent with the findings from the FACS sorting that there were similar amounts of GFP^+^ cells in the OL-Ribo-TRAP lines (Figure 2C). In contrast to the dramatic reduction in GFP^+^ OPCs from brains of 2aOKO-OPC-Ribo-TRAP mice as quantified by FACS sorting, the total numbers of Pdgfra^+^ cells as analyzed by IF were similar in 2a*^fl/fl^* and 2aOKO brains (Figure 2D). In addition, total numbers of NG2^+^ cells were also found to be similar in 2a*^fl/fl^* and 2aOKO brains (Supplemental Figure 5). Interestingly, the morphology of Pdgfra^+^ cells in 2aOKO brains was distinct from that of 2a*^fl/fl^* controls — with the majority of cells in the 2a*^fl/fl^* mouse brain exhibiting bipolar morphology typical of classical OPCs, whereas Pdgfra^+^ cells in the 2aOKO brain displayed a more complex and stunted morphology common to COPs and NFOLs (Figure 2E and Supplemental Figure 6). Given these stark changes in cell morphology, the disparity in cell numbers between GFP^+^ OPCs and Pdgfra^+^ OPCs is likely due to the fact that the Pdgfra-EGFP/Rpl10a line is reporting on classical Pdgfra^+^ OPCs and not NFOLs, a cell state in which Pdgfra continues to be expressed. Taken together, these data indicate that Mfsd2a deficiency in OPCs did not affect the total numbers of cells constituting the oligodendrocyte lineage, but rather suggest that Mfsd2a controls the stage of the oligodendrocyte lineage.

### Single-cell RNA-Seq of 2aOKO early postnatal brain reveals heterogeneity associated with OPC and immature oligodendrocyte development.

**Figure 3:**
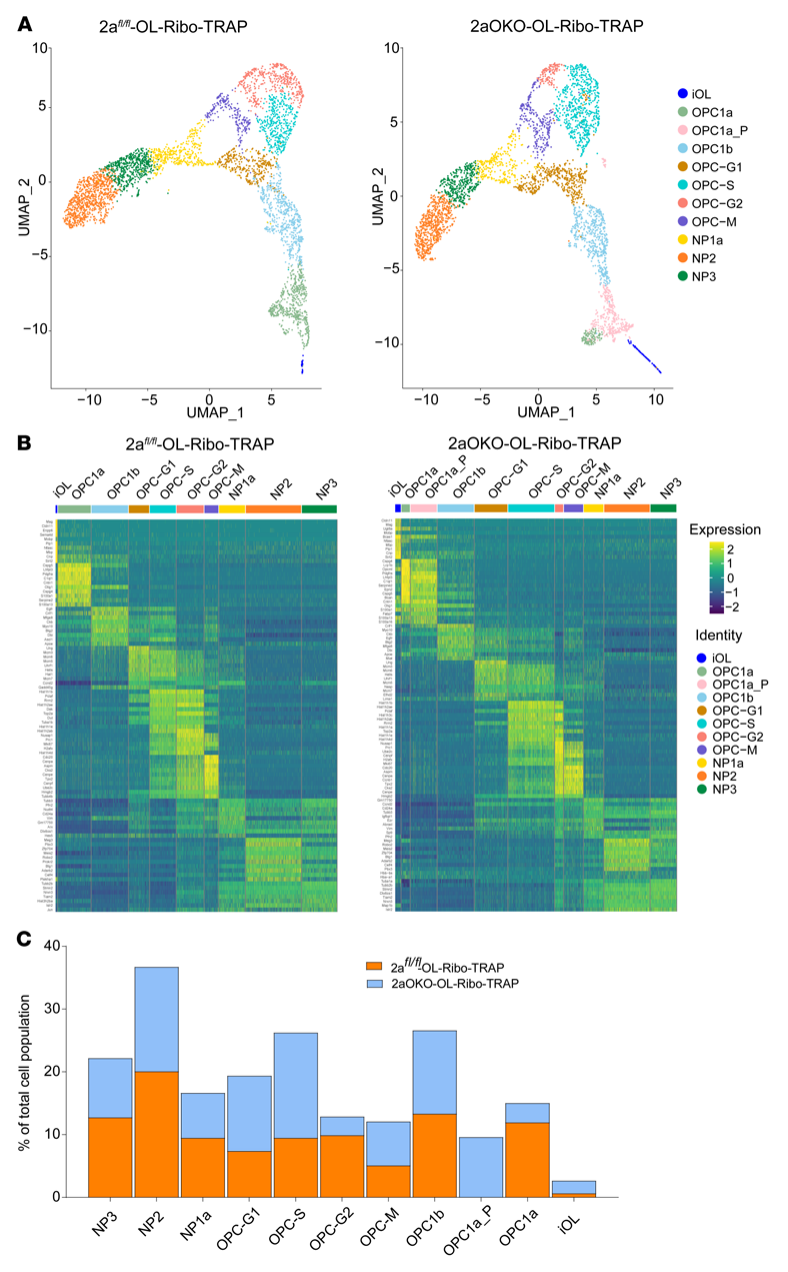
scRNA-Seq of 2aOKO early postnatal brain reveals altered population dynamics in the oligodendrocyte lineage. (**A**) Uniform manifold approximation and projection (UMAP) plots depicting the various oligodendrocyte lineage clusters of GFP^+^ flow-sorted cells from P8 brains of 2a*^fl/fl^*-OL-Ribo-TRAP (4,276 cells) and 2aOKO-OL-Ribo-TRAP (3,752 cells) mice. (**B**) Heatmap (green = low, yellow = high) of the top differentially expressed genes per cell cluster for both 2a*^fl/fl^*-OL-Ribo-TRAP and 2aOKO-OL-Ribo-TRAP brains. (**C**) Stacked bar plots show the percentage of each individual cell cluster relative to the total cell population for both 2a*^fl/fl^*-OL-Ribo-TRAP and 2aOKO-OL-Ribo-TRAP brains.

To determine the impact of OPC-specific deficiency of Mfsd2a on oligodendrocyte development, as well as the reasons underlying the differences in GFP^+^ and Pdgfra^+^ OPC population profiles between 2aOKO-Ribo-TRAP and 2aOKO brains, we performed 10x Genomics 3′ single-cell RNA-Seq (scRNA-Seq) on 4,276 and 3,752 FACS-sorted GFP^+^ cells representing the whole oligodendrocyte lineage obtained from P8 2a*^fl/fl^*-OL-Ribo-TRAP and 2aOKO-OL-Ribo-TRAP mouse brains, respectively. Clustering analysis based on the top 1,000 variable genes using a Seurat pipeline revealed 10 and 11 distinct clusters from 2a*^fl/fl^*-OL-Ribo-TRAP and 2aOKO-OL-Ribo-TRAP brain samples, respectively (Figure 3A). Marker gene expression analysis from each cluster identified pre-OPC neural progenitors (NP3, NP2, and NP1a); a population of cycling OPCs representing each cell cycle phase (OPC-G1, OPC-S, OPC-G2, and OPC-M); and 2 OPC clusters (OPC1b and OPC1a), with a unique cluster found only in 2aOKO (designated as OPC1a_P); as well as immature oligodendrocytes (iOLs) (Figure 3B and Supplemental Figure 3A). Partition-based graph abstraction (PAGA) as well as Slingshot-based pseudotime analysis showed the linear progression of oligodendrocyte lineage development in the following order: pre-OPC neural progenitors, cycling OPCs, OPC1b, OPC1a_P in 2aOKO only, OPC1a, and iOLs (Supplemental Figure 3, B and C). Consistent with previous reports, we identified Mfsd2a expression mainly in OPC1b, OPC1a, and iOL populations (Supplemental Table 1) (10, 11, 29). We were able to subdivide the OPC1a cluster from 2aOKO-OL-Ribo-TRAP brain into an additional cluster we termed OPC1a_P because cells in this cluster relative to OPC1a in 2a*^fl/fl^*-OL-Ribo-TRAP controls and/or 2aOKO-OL-Ribo-TRAP mice expressed higher levels of pre-OPC neural progenitor markers such as Stmn2, Tubb3, Dlx6os1, Arx, and Dlx1; and lower levels of classical OPC markers, namely Cspg4, Pdgfra, Pcdh15, and Ptprz1, and markers for iOLs, namely Gpr17, Bmp4, Neu4, and ITPR2 (Figure 3B) (10, 11, 29). Importantly, the OPC1a cluster constituted 12% of the total cell population found in 2a*^fl/fl^*-OL-Ribo-TRAP but only 3% of the total cell population in 2aOKO-OL-Ribo-TRAP, while the OPC1a_P cluster formed the majority of the OPC1a population, at 9.6% (Figure 3C). The seemingly inconsistent finding of dramatically reduced numbers of GFP^+^ cells together with similar levels of Pdgfra^+^ cells in 2aOKO-OL-Ribo-TRAP relative to control brains can be explained by the reduction in the OPC1a cluster (classical OPCs), with a proportional increase in cells that constitute the OPC1a_P cluster that are also Pdgfra^+^ (Figure 3C).

Notably, the iOL cluster was increased by approximately 2.8-fold in 2aOKO-OL-Ribo-TRAP compared with 2a*^fl/fl^*-OL-Ribo-TRAP (Figure 3, A and C), indicating that the progression of OPC differentiation is substantially increased in 2aOKO relative to control brains. In addition, the percentage of OPCs in the oligodendrocyte lineage in the cell cycle was increased by approximately 25%, with a corresponding approximately 25% decrease in the pre-OPC neural progenitor population. This finding again indicates a rapid progression along the various OPC developmental stages, leading to increased differentiation as a result of OPC-specific Mfsd2a deficiency (Figure 3, A and C). To directly assess this idea, we pulse-labeled 2aOKO and 2a*^fl/fl^* control mice with EdU and collected brains for analysis at 3 and 24 hours to quantify early (DNA synthesis) and late (mitosis and exit) steps in the cell cycle, respectively. Detection of EdU^+^Ki-67^+^ and EdU^+^Olig2^+^ cells on P8 mouse brain sections at these time points showed that indeed cell proliferation of the Olig2^+^ population was significantly higher in 2aOKO than 2a*^fl/fl^* control brains even though the cell cycle exit was not greatly altered (Supplemental Figure 4, A and B).

### OPC-specific Mfsd2a deficiency affects immature and mature oligodendrocyte population progression.

**Figure 4:**
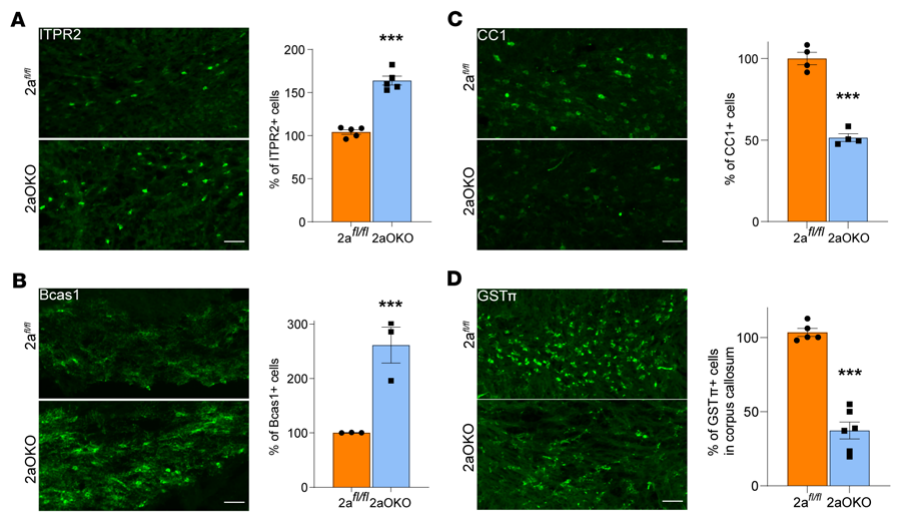
OPC-specific Mfsd2a deficiency affects immature and mature oligodendrocyte population progression. (**A**–**D**) IF analysis of P8 brain coronal sections with ITPR2 (**A**) and Bcas1 (**B**) antibodies indicates a significant reduction in numbers of iOLs at the corpus callosum in 2aOKO compared with 2a*^fl/fl^* control mice. *n* = 3–5 per genotype. MOLs are substantially reduced in corpus callosum of P8 brain from 2aOKO relative to 2a*^fl/fl^* control mice as determined by IF on coronal sections with CC1 (**C**) and GST_π_ antibodies (**D**). *n* = 4–6 per genotype. Scale bars: 50 μm. Data are presented as mean ± SEM; *n* = 4–6 per genotype. ***P < 0.0001 by 2-tailed Student’s *t* test (unpaired).

To confirm the large increase in the population of iOLs in 2aOKO brains as identified through scRNA-Seq and understand the impact on MOL development, we performed IF analysis for stage-specific markers on P8 brains. The ITPR2 marker, which mainly labels COPs/NFOLs (11), revealed an approximately 60% increase in this population in the corpus callosum of 2aOKO relative to 2a*^fl/fl^* control brains (Figure 4A). Using the Bcas1 antibody, which labels COPs/NFOLs and MFOLs but not MOLs (33), revealed that 2aOKO brains exhibited an approximately 2.5-fold increase in Bcas1^+^ cells in the corpus callosum relative to 2a*^fl/fl^* controls (Figure 4B). Quantifying the MOL population with CC1 antibody revealed a decrease by approximately 50% in 2aOKO compared with 2a*^fl/fl^* control corpus callosum (Figure 4C). Moreover, verifying this finding with another MOL marker, GST_π_, also showed an approximately 60% decrease in 2aOKO relative to 2a*^fl/fl^* control corpus callosum (Figure 4D). These data support the conclusion that loss of Mfsd2a in OPCs results in precocious differentiation of OPCs into committed but nonmyelinating oligodendrocytes, with a negative impact on the ability of COPs/NFOLs to mature into myelinating cell populations.

### OPC-specific Mfsd2a deficiency alters gene expression profiles in OPC and iOL populations.

**Figure 5:**
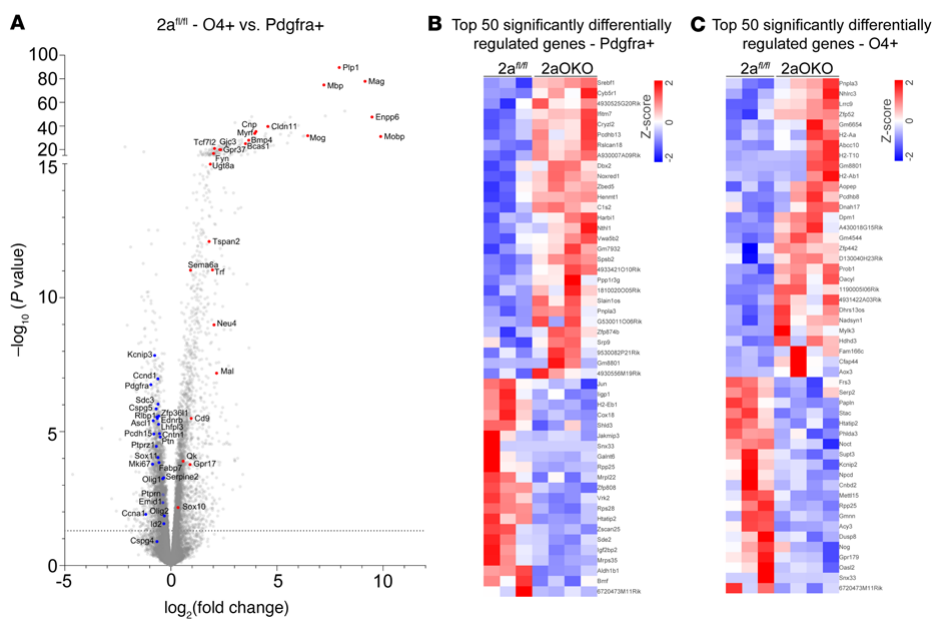
OPC-specific Mfsd2a deficiency alters gene expression profiles in OPC and iOL populations as determined by RNA-Seq. (**A**) Volcano plot shows transcriptional profile changes associated with differentiation of OPCs to iOLs in P8 brains of 2a*^fl/fl^* controls. Classical markers of MOLs and genes associated with cell differentiation are upregulated (red) and OPC markers are downregulated (blue) in O4^+^ cells (iOLs) compared with Pdgfra^+^ cells (OPCs). (**B**) Heatmap depicting the top 50 differentially expressed genes in OPCs from P8 brains of 2aOKO relative to 2a*^fl/fl^* control mice. (**C**) Heatmap displaying the top 50 differentially expressed genes in iOLs from P8 brain of 2aOKO compared with 2a*^fl/fl^* control mice. Color bars indicate *z* score transformation on median ratio–normalized counts. *n* = 3–4 per genotype.

To delineate the developmental differences associated with OPCs and iOLs due to loss of Mfsd2a, we isolated these cell populations by magnetic-activated cell sorting (MACS) using Pdgfra (for OPCs) and O4 (for iOLs) antibody microbeads, respectively, and performed bulk RNA-Seq on these populations. Differential gene expression analysis of OPCs (Pdgfra^+^) and iOLs (O4^+^) from 2a*^fl/fl^* controls showed that these cell populations were distinct, as evidenced by significant increases in differentiation and MOL markers in the O4^+^ population — such as Mobp, Enpp6, Mag, and Plp1 — with a reciprocal decrease in oligodendrocyte precursor markers — namely Pdgfra, Cspg5, Ptprz1, Fabp7, and Ascl1 — compared with the Pdgfra^+^ population (Figure 5A). These findings indicated that we isolated 2 oligodendrocyte cell populations having distinct differentiation states. Gene expression analysis of OPCs from 2a*^fl/fl^* and 2aOKO brains revealed a significant increase in Srebp-regulated genes involved in lipid metabolic pathways, namely Srebf1, Thrsp, Cyb5r1, and Tsku in 2aOKO OPCs (Figure 5B and Supplemental Table 2). Other lipid metabolic pathway genes, such as Cers3, Hpgds, and Acat3, were also upregulated in 2aOKO OPCs. Moreover, genes known to regulate OPC differentiation — Nkx6-1, Id2, Bmf, and Bdnf — were significantly downregulated in 2aOKO OPCs (34–37).

Similar to OPCs in 2aOKO brains, O4^+^ cells from 2aOKO brains exhibited upregulation of Srebp pathway genes such as Scd1, Pnpla3, and Tm6sf2 (Figure 5C and Supplemental Table 2). In addition, genes that are highly expressed at very late stages of oligodendrocyte differentiation, namely Mal, Apod, Mgst3, Acy3, Galnt6, Efna1, Tppp3, and Phlda3, were downregulated in O4^+^ cells from 2aOKO brains (Supplemental Table 2), consistent with a defect in maturation of iOLs (Figure 4) and reduced myelination (Figure 1 and Supplemental Figure 1, C and D). Interestingly, class II MHC genes, such as H2-Aa and H2-Ab1, that are known to be uniquely expressed in a subset of OPC populations identified in an experimental autoimmune encephalomyelitis mouse model (38), as well as the nonclassical MHC class I gene H2-T10, were significantly increased in O4^+^ cells from 2aOKO brains (Figure 5C). Several long noncoding RNAs, such as Gm8801, Slain1os, 1190005I06Rik, Uckl1os, and G530011O06Rik, that are expressed during various stages of oligodendrocyte development (39) were also differentially expressed in OPC and O4^+^ cells of 2aOKO brains (Supplemental Table 2), though their function in this process has yet to be clearly determined. Together with the results of the scRNA-Seq analysis, these data indicate that Mfsd2a deficiency in OPCs augments the rapid differentiation of OPCs into intermediate oligodendrocyte stages associated with altered gene regulation of multiple pathways including lipid metabolism and differentiation, while progression from iOLs into maturation states essential for proper myelination is hindered.

### OPC-specific Mfsd2a deficiency alters lipid composition in OPC and iOL populations.

**Figure 6:**
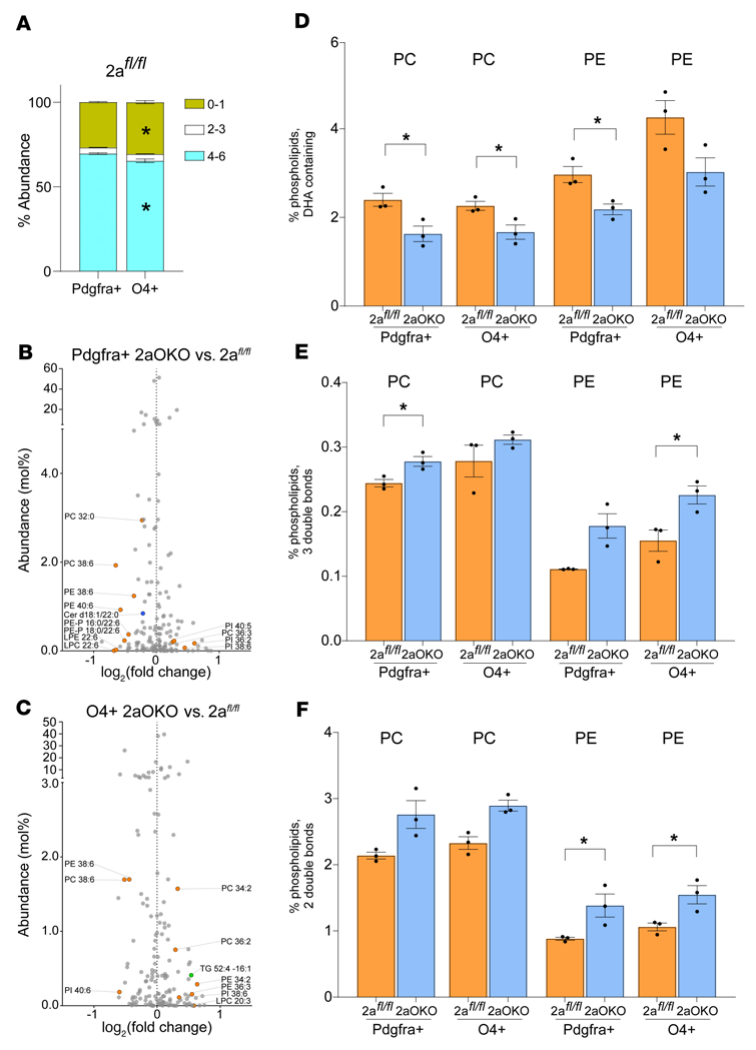
OPC-specific Mfsd2a deficiency alters lipid composition in OPC and iOL populations. Lipidomic analysis of OPCs (Pdgfra^+^) and iOLs (O4^+^) isolated from P8 brains of 2a*^fl/fl^* and 2aOKO mice. (**A**) Stacked bar plots show the percent distribution of phospholipids with different degrees of unsaturation. (**B**) Volcano plot showing the percentage of total lipids that are significantly different in OPCs from 2aOKO relative to 2a*^fl/fl^* control brains. Significantly different phospholipids are indicated in orange and ceramides in blue. (**C**) Volcano plot showing the percentage of total lipids that are significantly different in iOLs from 2aOKO relative to 2a*^fl/fl^* control brains. Significantly different phospholipids are indicated in orange and triglycerides in green. (**D**–**F**) Percentage of PC and PE containing DHA or fatty acids with a total of 2 or 3 double bonds in OPCs and iOLs from 2aOKO and 2a*^fl/fl^* control brains. Data are presented as mean ± SEM; *n* = 3 per genotype. *P < 0.05 by 2-tailed Student’s *t* test (unpaired).

To identify the changes in lipid composition as OPCs differentiate into iOLs, we isolated these cells types (Pdgfra^+^ and O4^+^ cells) from 2a*^fl/fl^* and 2aOKO brains using the MACS approach described above. Lipidomics analysis using targeted mass spectrometry (MS) showed that OPCs from 2a*^fl/fl^* controls were significantly enriched in the percentage of phospholipids with polyunsaturated fatty acyl chains (PUFAs) containing 4–6 double bonds relative to iOLs, while iOLs were enriched in the percentage of saturated and monounsaturated phospholipid content relative to OPCs (Figure 6A). Phospholipids containing fatty acyl chains having 2–3 double bonds were largely similar in these distinct cell populations. Thus, as OPCs differentiated into mature subtypes, their phospholipid pools transitioned from being enriched in PUFAs to having a more saturated fatty acid composition. Remarkably, the lipid profiles of OPCs and iOLs in 2aOKO brains showed a significant reduction in PC and PE species containing DHA (Figure 6, B–D), consistent with the reduced transport of DHA due to the absence of Mfsd2a in these cells. In contrast, OPCs and iOLs from 2aOKO brains had significantly increased PC and PE species having 2–3 double bonds, which are known products of de novo fatty acid synthesis controlled by Srebp-1 (Figure 6, B, C, E, and F). These data indicate that loss of Mfsd2a resulted in reduced uptake of DHA-containing phospholipid species, which leads to a compensatory increase in synthesis of unsaturated fatty acids containing 2 and 3 double bonds. Overall, altered lipid composition and associated gene expression changes correlate with disruption in the normal progression of oligodendrocyte development, leading to hypomyelination.

## Discussion

Myelination of neuronal axons is a complex process carried out by oligodendrocytes, whose development follows a highly ordered pathway from the precursor cell to the mature myelinating stage (10, 11). Disruption along any of these steps can lead to deleterious consequences ranging from oligodendrocyte death to hypomyelination to precocious hypermyelination (40). As evidenced from the literature, developmental regulation of oligodendrocyte lineage through intrinsic factors regulating gene expression is relatively well characterized, as are some of the main extrinsic signals derived from the extracellular environment, such as growth factors and neural activity (41–43). However, relatively little is known regarding the influence of lipids on oligodendrocyte development. Here, we report that the LPC transporter Mfsd2a is crucial for regulating the phospholipid composition of OPCs and oligodendrocyte cell state, which points to polyunsaturated LPC species transported by Mfsd2a as extrinsic factors influencing oligodendrocyte development. Loss of Mfsd2a in OPCs led to the precocious progression of OPCs into an iOL state that correlated with reduced DHA incorporation in phospholipid species, resulting in the depletion of the OPC pool and incomplete transitioning from immature oligodendrocytes into MOLs, ultimately resulting in postnatal brain hypomyelination.

One of the prominent brain phenotypes of Mfs2a deficiency induced at either the BBB or conventionally in the entire mouse is microcephaly and hypomyelination (17, 28) (Supplemental Figure 1A), similar to that in humans with loss-of-function mutations in Mfsd2a. It was not understood whether hypomyelination was a primary or secondary effect of reduced brain growth resulting from the absence of LPC uptake into the brain. We were able to separate these 2 brain phenotypes by deletion of Mfsd2a specifically in OPCs, which did not affect brain growth but did result in hypomyelination, implying that LPC transport into OPCs is critical for myelination. That Mfsd2a is specifically expressed by OPCs suggests that LPCs are found in the interstitium of the brain and could be carried by apolipoproteins such as apoE and apoD, analogous to their transport by albumin in blood. Although the brain interstitial lipidome remains to be elucidated, our findings highlight the importance of its functional role in myelination.

Consistent with recent scRNA-Seq results from P7 brain samples (10), we were able to identify the major cell type clusters using scRNA-Seq of OL-Ribo-TRAP mice. Moreover, we were able to distinguish additional clusters for cycling OPCs representing each cell cycle phase, but only a single cluster of iOLs. Differential gene expression as well as cluster analysis of samples from 2a*^fl/fl^*-OL-Ribo-TRAP and 2aOKO-OL-Ribo-TRAP mice showed that clusters pertaining to specific subtypes of oligodendrocyte lineage development were similar in the 2 groups. However, major differences were found in the percentage of total cell population represented by each subtype — particularly OPC1a and iOL as well as the oligodendrocyte lineage progression, indicating that Mfsd2a was regulating cell state and not cell survival. It should be noted that Mfsd2a expression was more prominent only in OPC1b, OPC1a, and iOL clusters (10, 11, 29) (Supplemental Table 1); thus, any impact of gene-targeted deletion of Mfsd2a on cell populations from earlier phases of the oligodendrocyte lineage — neural progenitors or cycling OPCs — would most likely be due to adaptive changes in lieu of the transition of the OPC1a cell population to COPs/NFOLs. We identified a unique OPC cluster that we designated OPC1a_P only in 2aOKO brains that expressed higher levels of markers from pre-OPCs in addition to lower levels of classical OPC1a and iOL markers, indicating that gene expression and regulation are differentially altered in response to Mfsd2a deficiency in OPCs. As observed in pseudotime analysis, it is predicted that OPC1a_P could progress into a classical OPC state, undergo rapid differentiation directly into iOLs, or exist as an altogether unique cell population. Most strikingly, 2aOKO brains exhibited a dramatic increase in iOL population, indicating precocious differentiation of OPCs that correlated with defective oligodendrocyte maturation and myelination.

Our laboratory has previously shown using multiple Mfsd2a-KO models that reduced DHA transport into the brain increased de novo lipogenesis through activation of the Srebp-1 pathways (28). Transcriptomics on isolated OPCs and iOLs also supported this finding, wherein Srebp-1–regulated lipid metabolic pathway genes were significantly upregulated in OPCs from 2aOKO mice. A previous study using lipidomics on primary rat OPCs in culture found that OPCs differentiated into MOLs showed a distinct increase in saturated fatty acid levels, with a decrease in polyunsaturated fatty acid content relative to that in OPCs (44), suggestive of higher activation of Srebp-1 in MOLs than in OPCs. In agreement with this study, we found that control OPCs had a higher percentage of unsaturated fatty acids in phospholipid species relative to iOLs. Also consistent with the function of Mfsd2a as an LPC-DHA transporter, levels of DHA-containing species were reduced in OPCs and iOLs of 2aOKO brains, with a concomitant increase in monounsaturated fatty acids, which are products of the Srebp-1 pathway. Although Mfsd2a is a lysolipid transporter that has direct effects on phospholipid membrane composition, it is not known how membrane phospholipid fatty acid composition and Srebp-1 regulation of gene targets might converge to synchronize oligodendrocyte differentiation and myelination. Further work is required to determine the molecular mechanism linking lipidomic changes consequent to Mfsd2a deficiency with oligodendrocyte cell state and function. In summary, our findings reveal that LPCs can be considered as extrinsic factors that maintain the OPC state and contribute to the physiological regulation of OPC differentiation. The function of the Mfsd2a/LPC pathway in myelination could be relevant in light of the recent findings that Mfsd2a is downregulated in aging, wherein brain myelination and myelin repair are known to be reduced (45). It will be important to determine whether the Mfsd2a/LPC pathway plays a similar role in adult OPC differentiation and myelination and whether LPC supplementation could help to augment remyelination in demyelinating diseases such as multiple sclerosis and in normal aging.

## Methods

### Animal experimental procedures.

C57BL/6-Tg (Pdgfra-cre)1Clc/J (Pdgfra-cre, stock 013148) (46), B6;CBA-Tg(Sox10-cre)1Wdr/J (Sox10-cre, stock 025807) (47), B6.Cg-Tg(Plp1-cre/ERT)3Pop/J (Plp1-ERT-cre, stock 005975) (48), B6;FVB-Tg(Olig2-EGFP/Rpl10a)JD97Htz/J (Olig2-Ribo-TRAP, stock 030265) (31), B6;FVB-Tg(Pdgfra-EGFP/Rpl10a)JD340Htz/J (Pdgfra-Ribo-TRAP, stock 030268) (32) were purchased from the Jackson Laboratory. All the mouse strains mentioned above were crossed with a mouse strain carrying an Mfsd2a floxed allele (2a*^fl/fl^*) to generate homozygous strains for this allele. Generation of Mfsd2a-KO mice (2aKO mice) and the 2aKI-ERT2-cre: tdtomato reporter mouse line was described previously (18, 30). Mice were housed in a 12-hour light/12-hour dark cycle with controlled humidity and temperature at 23°C, fed ad libitum on normal chow diet (Global 18% Protein Rodent Diet from Harlan, Envigo), and had free access to water. Tamoxifen-induced cre recombination was carried out by intraperitoneal injection of 200 mg/kg body weight tamoxifen (MilliporeSigma, T5648) dissolved in corn oil. Tamoxifen was administered as 3 injections spaced 24 hours apart starting with P3 pups for tdtomato reporter experiments or only a single dose with P5 Plp1-cre/ERT pups, and tissue samples were collected 5 or 3 days after last injection, respectively. For EdU experiments, P7 or P8 pups were given 1 intraperitoneal injection of 250 μg 5-ethynyl-2′-deoxyuridine (EdU, MilliporeSigma, 900584) dissolved in DMSO as indicated. Mice pups were anesthetized with a combination of ketamine (20 mg/kg body weight) and xylazine (2 mg/kg body weight) and perfused transcardially with 1× PBS and 4% paraformaldehyde (PFA) in 1× PBS prior to tissue harvest.

### Immunohistochemistry and quantification.

Brain tissues harvested after perfusion were fixed in 4% PFA for 1 hour at 4°C, cryoprotected in 30% sucrose in 1× PBS for 48 hours at 4°C and embedded in OCT (Sakura Finetek USA). Cryosections (20 μm) in the coronal plane were prepared using Leica Cryostat CM1520. For EdU-labeled brain samples, EdU detection on cryosections (Click-iT EdU Imaging Kit, Invitrogen, C10340) was performed according to the manufacturer’s instructions and then processed as mentioned below for immunohistochemistry. After antigen heat retrieval using sodium citrate buffer, cryosections were incubated in blocking buffer (10% normal goat serum, 1% BSA, 0.3 M glycine in PBS–0.1% Triton-X) for 1 hour at room temperature. Primary and secondary antibodies were prepared in blocking buffer. Sections were incubated overnight at 4°C with the following primary antibodies: Pdgfra (1:100, Cell Signaling Technology, 3174), MBP (1:200, Bio-Rad, MCA409S), MAG (1:100, MilliporeSigma, MAB1567), CNPase (1:300, Cell Signaling Technology, 5664), GFP (1:500, Cell Signaling Technology, 2555), Olig2 (1:200, MilliporeSigma, MABN50), Ki67 (1:400, Abcam, ab15580), ITPR2 (1:40, MilliporeSigma, AB3000), Bcas1 (1:1000, Santa Cruz Biotechnology Inc., sc-136342), CC1 (1:50, Abcam, ab16794), GST_π_ (1:100, BD Transduction Laboratories, 610718). Sections were then incubated with Alexa Fluor 488/568/647 secondary antibodies (1:500, Invitrogen) and nuclei were counterstained with Hoechst 33342 (1:1,000, Invitrogen). After final washing steps with PBS-T, sections were mounted using Fluoromount-G (Southern Biotech, 0100-01) and images were obtained using an LSM710 Confocal Microscope (Zeiss). Immunolabeled cells in the corpus callosum region were counted manually. Sholl analysis to assess the morphological complexity of Pdgfra^+^ OPCs was performed using the Simple Neurite Tracing Plugin from Fiji (ImageJ).

### Electron microscopy analysis.

Mice were anesthetized as indicated above and transcardially perfused with 0.1 M cacodylate buffer, followed by a fixative solution containing 2% PFA and 2.5% glutaraldehyde in 0.1 M cacodylate buffer. Brain tissues harvested after perfusion were immersed in the same fixative solution overnight at 4°C, and corpus callosa were isolated from 1 mm coronal slices of brain and postfixed in 2% osmium tetroxide. Samples were rinsed, dehydrated in a graded series of ethanol, and embedded in epoxy resin. Ultrathin sections were prepared and stained with 1% uranyl acetate and lead citrate. Stained sections were examined using a JEOL JEM-1400Flash TEM. A minimum of 200 myelinated axons were counted per animal, and the g-ratio of axons was calculated by determining the ratio of the inner diameter of the axon to the outer diameter of the axon fiber (axon and myelin).

### FACS sorting.

Cerebrum from Ribo-TRAP mouse brain models was excised, and dissociation was performed with a Papain Neural Tissue Dissociation Kit (Miltenyi Biotec, 130-092-628) following the manufacturer’s instructions. A single-cell suspension was then FACS sorted for GFP^+^ cells using a BD FACSAria II SORP Sorter (BD Biosciences), and the percentage of GFP^+^ cells relative to the total cell population was plotted as graphs. For scRNA-Seq, GFP^+^ cells were collected in PBS/0.2% BSA buffer before further processing.

### scRNA-Seq.

FACS-sorted GFP^+^ cells from 2a*^fl/fl^*-OL-Ribo-TRAP control and 2aOKO-OL-Ribo-TRAP mouse brains (1,000 cells per μL) were processed using a Chromium Single Cell 3′ kit v3 (10x Genomics, PN-1000092) for the generation of single-cell Gel Bead-In-Emulsion (GEM) capture, barcoding, reverse transcription, cDNA amplification, and library construction according to the manufacturer’s guidelines. Libraries were sequenced on the Illumina HiSeq 4000 platform.

### Single-cell data analysis.

Raw reads in the FASTQ files were quantified via transcript-associated unique tags (unique molecular identifiers [UMIs]) using Cell Ranger (v.3.1.0) and aligned to the mouse reference genome (mm10-3.0.0), with the expected cell recovery per sample set to 10,000. The data output across all samples was comparable, with the estimated number of cells ranging from 9,526 to 10,084, with a median of 2,540–2,565 genes per cell, a mean count of 49,239–49,453 reads per cell across samples, and a unique read mapping rate of 91.7%–92.2%. Downstream data analysis was performed using the Seurat package (v.4.0.2) (49) and customized scripts in R. The Cell Ranger–derived raw expression matrix was normalized and log transformed by a scale factor of 10,000 using the *NormalizeData* function in Seurat. Cells with UMI counts between 3,000 and 30,000 for 1,200 or more genes and with less than 15% mitochondrial genes were retained for further analysis. Similarly, genes with average log_10_ UMI count of –2 or higher and with 2 or more UMIs observed in 5 or more cells were retained. Additionally, cells not expressing any of the following cell type–specific marker genes (endothelium — Cldn5, Ly6c1, Vtn, Col4a1, Igfbp7; astrocytes — Agt, Aqp4, Gja1, Htra1; microglia — Hexb, Cx3cr1, Ctss, C1qa, C1qb; neurons — Neurod1, Pcp4, Calb2, Eomes, Sema3c) were removed. Cells that expressed one of the early markers Bmp4, Itpr2, Cnksr3, and Neu4 together with one of the late markers Ctps, Mog, and Klk6 were also filtered out, resulting in a final tally of 4,276 cells in the 2a*^fl/fl^*-OL-Ribo-TRAP sample and 3,752 cells in 2aOKO-OL-Ribo-TRAP samples for downstream analysis. The top 2,000 highly variable genes were screened by the *FindVariableFeatures* function via the “vst” method. Expression data were prepared for principal component analysis (PCA) after linear scaling by using the *ScaleData* function. PCA was performed with the *RunPCA* function based on the most variable 2,000 genes. Graph-based clustering was performed in Seurat by first constructing a k-nearest neighbor graph using the *FindNeighbors* function, followed by identifying the number of clusters via the *FindClusters* function with resolution 0.6, resulting in 10 and 11 clusters in 2a*^fl/fl^*-OL-Ribo-TRAP and 2aOKO-OL-Ribo-TRAP samples, respectively. Clusters were visualized in 2D space via the (uniform manifold approximation and projection (UMAP) method (50) based on the first 12 principal components, with minimum distance set to 0.3 and number of neighbors fixed at 30. Cluster-based differential gene expression was performed by comparing each cluster to others using the *FindAllMarkers* function in the Seurat package. For the inference of cell lineage structures, a pseudotemporal reconstruction for the inference of cell lineage structures was performed via the *slingshot* function in the *dynwrap* package (https://cran.r-project.org/web/packages/dynwrap/index.html).

### Transcriptomics.

Brain dissociation from 2a*^fl/fl^* and 2aOKO mouse brain was prepared as indicated above. Immunomagnetic cell sorting from single-cell suspensions for OPCs and iOLs was performed on LS columns (Miltenyi Biotec, 130-042-401) using microbead kits from Miltenyi Biotec (for OPCs: CD140a [PDGFRα] MicroBead Kit, mouse, 130-101-502; and for O4: anti-O4 microbeads, human, mouse, rat, 130-096-670) based on the manufacturer’s protocols. Total RNA was extracted using a QIAGEN RNeasy Mini kit, and 900 ng per sample was used for library preparation using NEBNext Ultra TEM RNA Library Prep Kit for Illumina (NEB) according to the manufacturer’s instructions and sequenced on an Illumina HiSeq2000. Partek Flow (version 10) was used for analysis, and paired-end sequenced reads were aligned to mm39 genome using STAR alignment 2.7.8a (51) and annotated using Ref-Seq. Features were filtered using recommended parameters and median ratio normalized. Differential gene expression analysis was performed using DESeq2 (52) and genes with fold change of at least 1.25 and *P* < 0.05 were considered significant.

### Lipidomics.

Isolation of OPCs and iOLs was performed as mentioned above. A single-phase butanol/methanol (1:1; BuMe) protocol was applied for lipid extraction. The BuMe solution contained a mixture of internal standards (ISTD), including acylcarnitine (AcylCarn 16:0 d3), dihydroceramide (Cer d18:0/8:0), ceramide (Cer d18:1/8:0), hexosylceramide (Hex1Cer d18:1/8:0), monosialodihexosylganglioside (GM3 d18:1/18:0 d3), sphinganine (Sph d18:0 d7), sphingosine (Sph d18:1 d7), sphingomyelin (SM d18:1/12:0), diacylglycerol (DG 30:0), triacylglycerol (TG 36:0), lysophosphatidylcholine (LPC 13:0), lysophosphatidylethanolamine (LPE 14:0), phosphatidylcholine (PC 26:0), phosphatidylethanolamine (PE 34:0), phosphatidylglycerol (PG 34:0), phosphatidylinositol (PI 25:0), and phosphatidylserine (PS 34:0). An aliquot of 200 μL BuMe solution was added into cell pellets. Samples were vortexed for 10 seconds and sonicated with ice in an ultrasonic bath for 40 minutes. Samples were centrifuged at 14,000 *g* for 10 minutes at room temperature, and then supernatants were transferred to the MS vial for liquid chromatography–MS (LC-MS) analysis.

The chromatographic separation was performed on an Agilent ZORBAX Eclipse Plus Rapid Resolution HD C18 (95 Å, 2.1 × 50 mm, 1.8 μm) column at 0.4 mL min^–1^ in an Agilent 1290 UHPLC system. The mobile phase A was 10 mM ammonium formate in 40% acetonitrile and 60% water, and the mobile phase B was 10 mM ammonium formate in 10% acetonitrile and 90% isopropanol. The gradient was set as 60% B from 0 to 2.00 minutes, 100% B from 2.00 minutes to 14.00 minutes, 100% B decreased to 20% until 14.01 minutes and 20% B continued until 15.8 minutes. The total run time was 15.80 minutes. The analysis was performed on an Agilent 6495 QQQ mass spectrometer (Agilent Technologies). The Agilent Jet Stream Electrospray Ionization (AJS ESI) source parameters were set as follows: dry gas temperature and flow were 250°C and 14 L min^–1^, respectively. Nebulizer pressure was 35 psi, and sheath gas temperature and flow were set to 250°C and 11 L min^–1^, respectively. Capillary voltage and nozzle voltage set to 3,000 V and 1,000 V, respectively, and the delta EMV was 200 V. Both positive and negative high/low pressure radio frequency were set to 150/60. The fragmentor voltage was 166 V, and the cycle time was 900 ms. Retention time windows, cell acceleration voltage, and collision energy varied depending on different lipid classes. For each analysis, 5 μL extract was injected. The MS was performed in positive ionization mode with a dynamic multiple reaction monitoring method (dMRM). Relative lipid concentrations in each sample were obtained after normalizing by MS peak area of lipid species, MS peak area of ISTDs, concentrations of ISTDs in BuMe solution, and cell number of the samples. Lipid species were categorized into phospholipids, sphingolipids, or neutral lipids and represented as mol% of lipid class.

### Statistics.

The numerical results are represented as mean ± SEM. Statistical differences were calculated using 2-tailed Student’s *t* test (unpaired), and graphs were prepared using GraphPad Prism 9.0. *P* values of less than 0.05 were considered to be significant in all cases. The numbers of animals used to perform the experiments are indicated in the figure legends.

### Study approval.

All mouse experiments were performed using protocols approved by SingHealth IACUC (protocol 2018/SHS/1416).

### Data availability.

scRNA-Seq and bulk RNA-Seq raw data files were deposited in the NCBI’s Gene Expression Omnibus (GEO GSE211879 and GSE211228). See Supporting Data Values for source data underlying reported values.

## Author contributions

All experiments and data analysis were performed by VS. Analysis of scRNA-Seq data was carried out by MH and VS with supervision by SG. Targeted lipidomics was performed by JO with supervision by FT and MRW. Analysis of RNA-Seq and lipidomics was assisted by BHW. Tissue isolation was assisted by DLG. The manuscript was written by VS and DLS. Figures were prepared by VS. Guidance, input, experimental design and analysis at all stages of the project were provided by DLS.

## Supplementary Material

Supplemental data

Supplemental table 1

Supplemental table 2

Supporting data values

## Figures and Tables

**Table 1:**
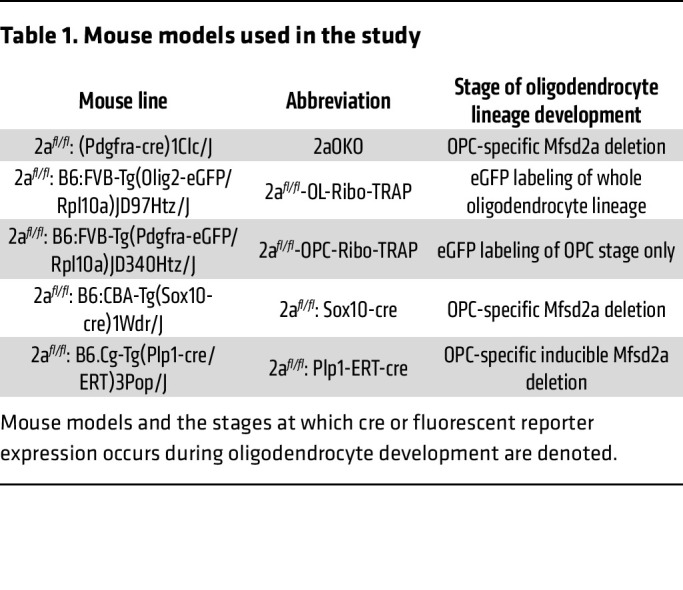
Mouse models used in the study

## Version 1 04/27/2023

In-Press Preview

## Version 2 06/15/2023

Electronic publication
