# Supplementary material for: Deficiency in the omega-3 lysolipid transporter Mfsd2a leads to aberrant oligodendrocyte lineage development and hypomyelination
Source: J Clin Invest. 2023 Jun 15;133(12):e164118. doi: 10.1172/JCI164118 (PMC10266779; doi:10.1172/JCI164118)
Supplement: Supplemental data [file jci-133-164118-s025.pdf]

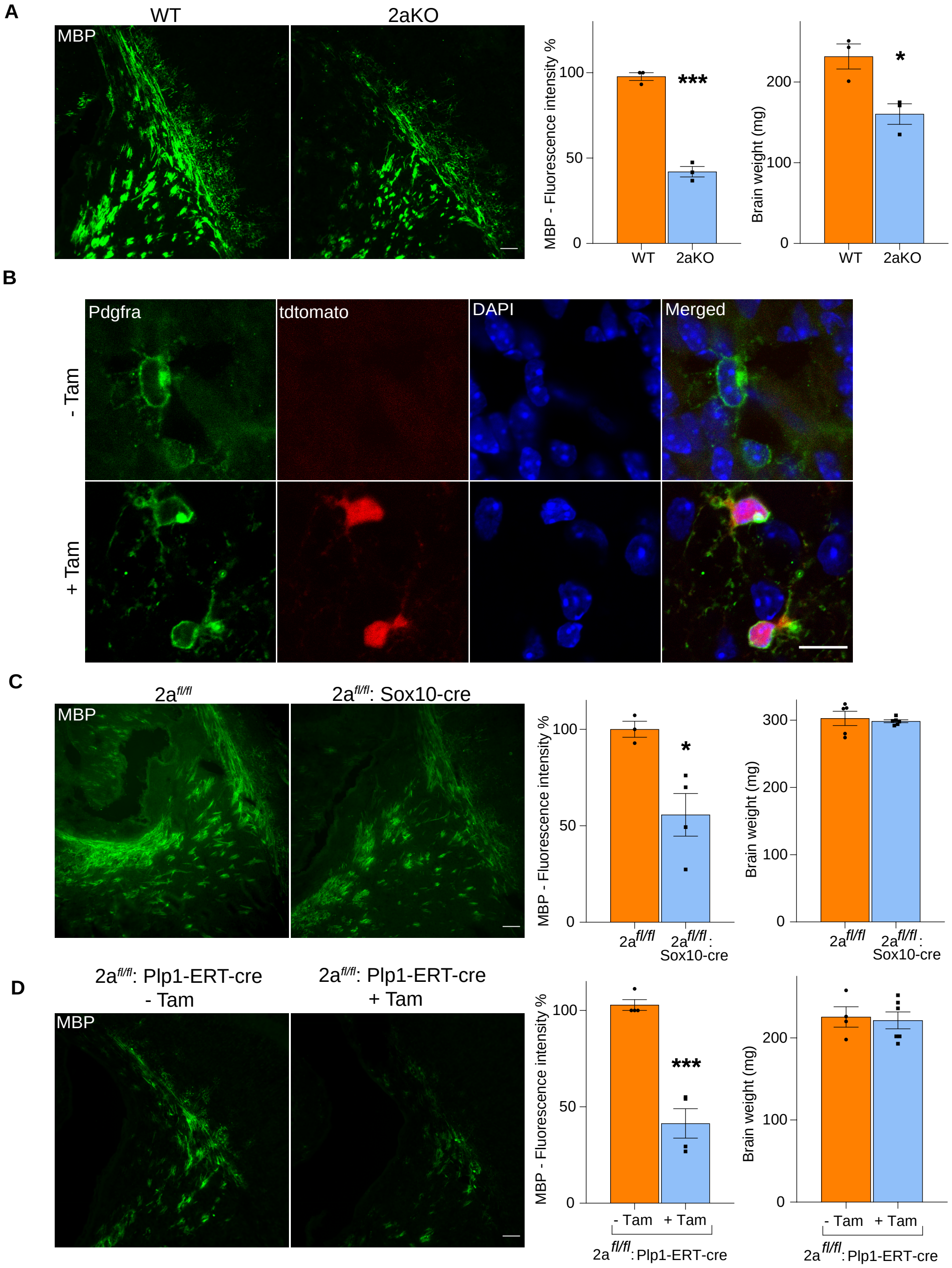

Fig S1. Conventional Mfsd2a deficiency and OPC-specific Mfsd2a deficiency causes hypomyelination. (**A**) Representative images and quantification of coronal sections from P8 WT and 2aKO brain immunostained with myelin marker protein (MBP) show postnatal hypomyelination in 2aKO. Reduced brain weights in 2aKO relative to WT indicates microcephaly. Data are represented as mean ± S.E.M, n = 3 per genotype, n = 3 per genotype. ***p < 0.0001, *p < 0.01 by 2-tailed Student's t-test (unpaired). (**B**). IF on coronal sections from P10 brain of tamoxifen treated (+Tam) 2aKI-ERT2-cre: tdtomato reporter line, immunostained with Pdgfra indicates expression of Mfsd2a in OPCs. Brain of untreated mice (-Tam) served as a negative control. Scale bar = 10µm. (**C**) Representative images and quantification of coronal sections from P8 brain of $2a^{fl/fl}$: Sox10-cre and $2a^{fl/fl}$ controls immunostained with MBP indicates hypomyelination of $2a^{fl/fl}$: Sox10-cre compared to $2a^{fl/fl}$ controls. (**D**) Representative images and quantification of coronal sections from P8 brains of $2a^{fl/fl}$:Plp-ERT-cre (+Tam) and $2a^{fl/fl}$: Plp-ERT-cre (-Tam) controls immunostained with MBP indicates hypomyelination after Mfsd2a knockout in OPCs. The area shown is the corpus callosum. Scale bar = 100µm. Brain weights from both (**C**) and (**D**) indicates no microcephaly. Data are represented as mean ± S.E.M, n = 3 - 6 per genotype. ***p < 0.0001, *p < 0.05 by 2-tailed Student's t-test (unpaired).

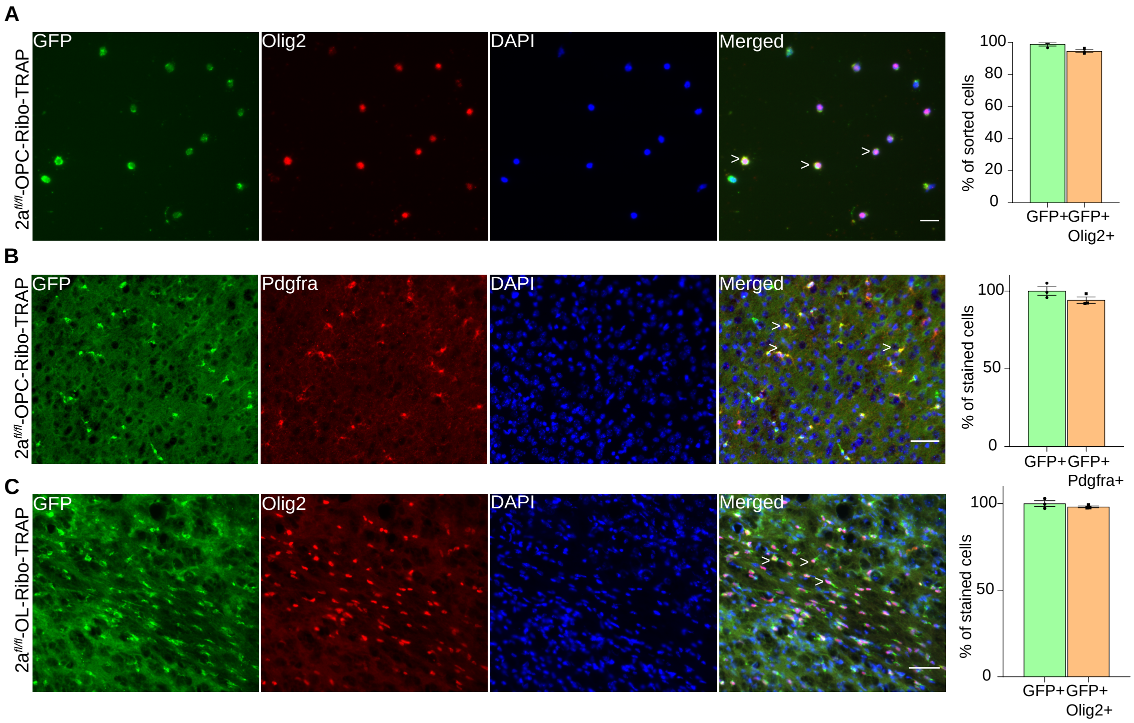

Fig S2. Ribo-TRAP mouse models can be used to isolate and analyse OPCs and total oligodendrocyte populations in mouse brain. (**A**) IF using GFP and Olig2 antibody on flow sorted GFP+ cells from P8 brain of 2a$^{fl/fl}$-OPC-Ribo-TRAP control indicate oligodendrocyte population can be isolated at ~95% purity, n = 3 per genotype. Scale bar = 20µm. (**B-C**) IF using GFP and Pdgfra or Olig2 antibody on coronal sections from P8 brain of 2a$^{fl/fl}$-OPC-Ribo-TRAP or 2a$^{fl/fl}$-OL-Ribo-TRAP indicate ~94% of GFP+ cells are OPCs (Pdgfra+) and ~98% of GFP+ cells are from the oligodendrocyte lineage (Olig2+). Colocalization of GFP+ cells with respective antibody markers probed are indicated with white arrows at the lateral corpus callosum and adjoining cortex in genu region of the brain. Scale bar = 50µm.

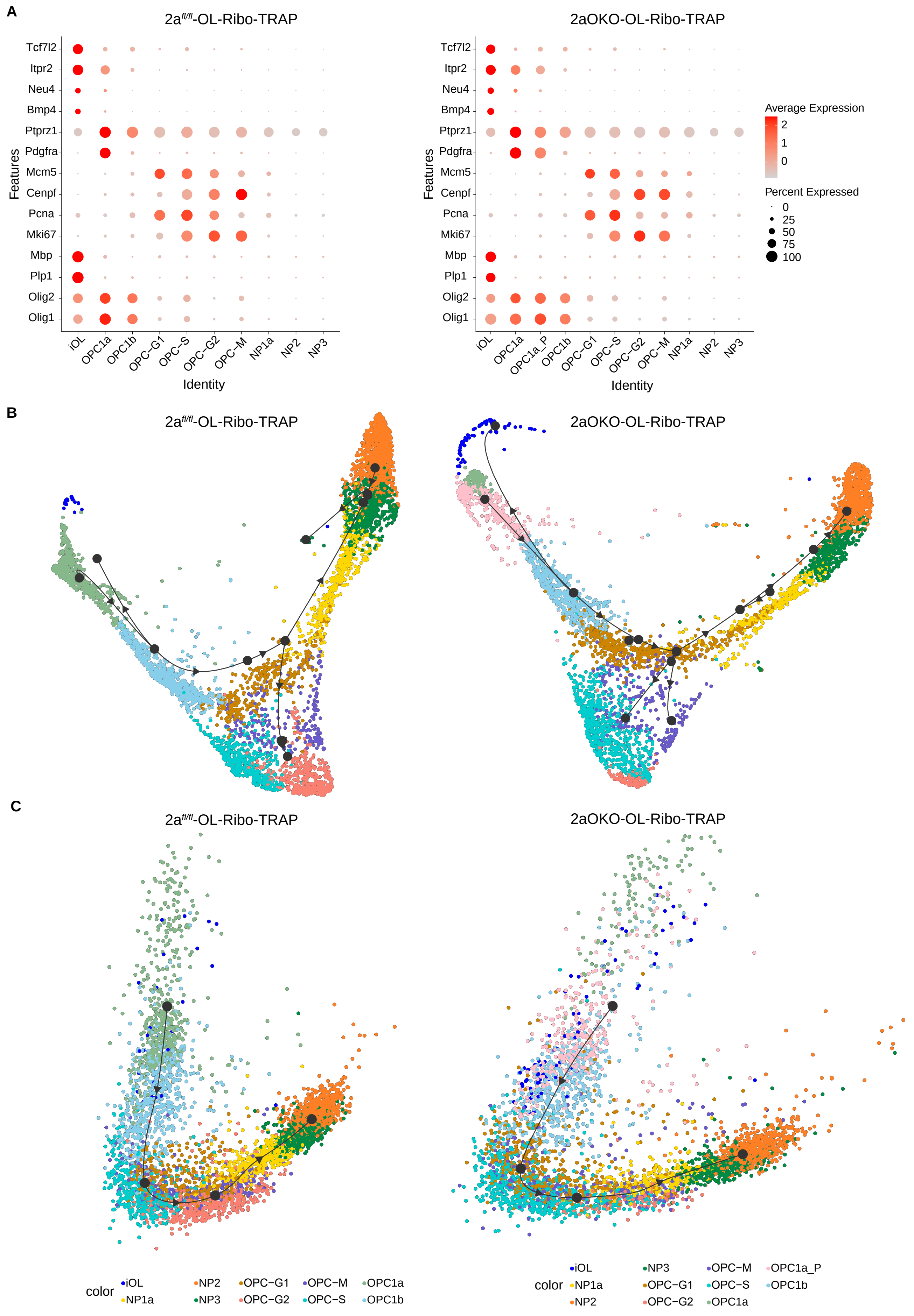

Fig S3. Single cell RNAseq of 2aOKO early postnatal brain reveals heterogeneity associated with OPC and immature oligodendrocyte development. (**A**) Dot plots show expression of classical markers from subtypes of the oligodendrocyte lineage identified by scRNA-Seq in P8 mouse brain of 2a$^{fl/fl}$-OL-Ribo-TRAP and 2aOKO-OL-Ribo-TRAP. Color scale indicates average expression, red = high, gray = low and size of dots denote percent expressed. (**B** - **C**) Pseudotime analysis using PAGA (**B**) and Slingshot (**C**) of all identified clusters from 2a$^{fl/fl}$-OL-Ribo-TRAP and 2aOKO-OL-Ribo-TRAP. Arrows mark the developmental progression of cells from all identified clusters.

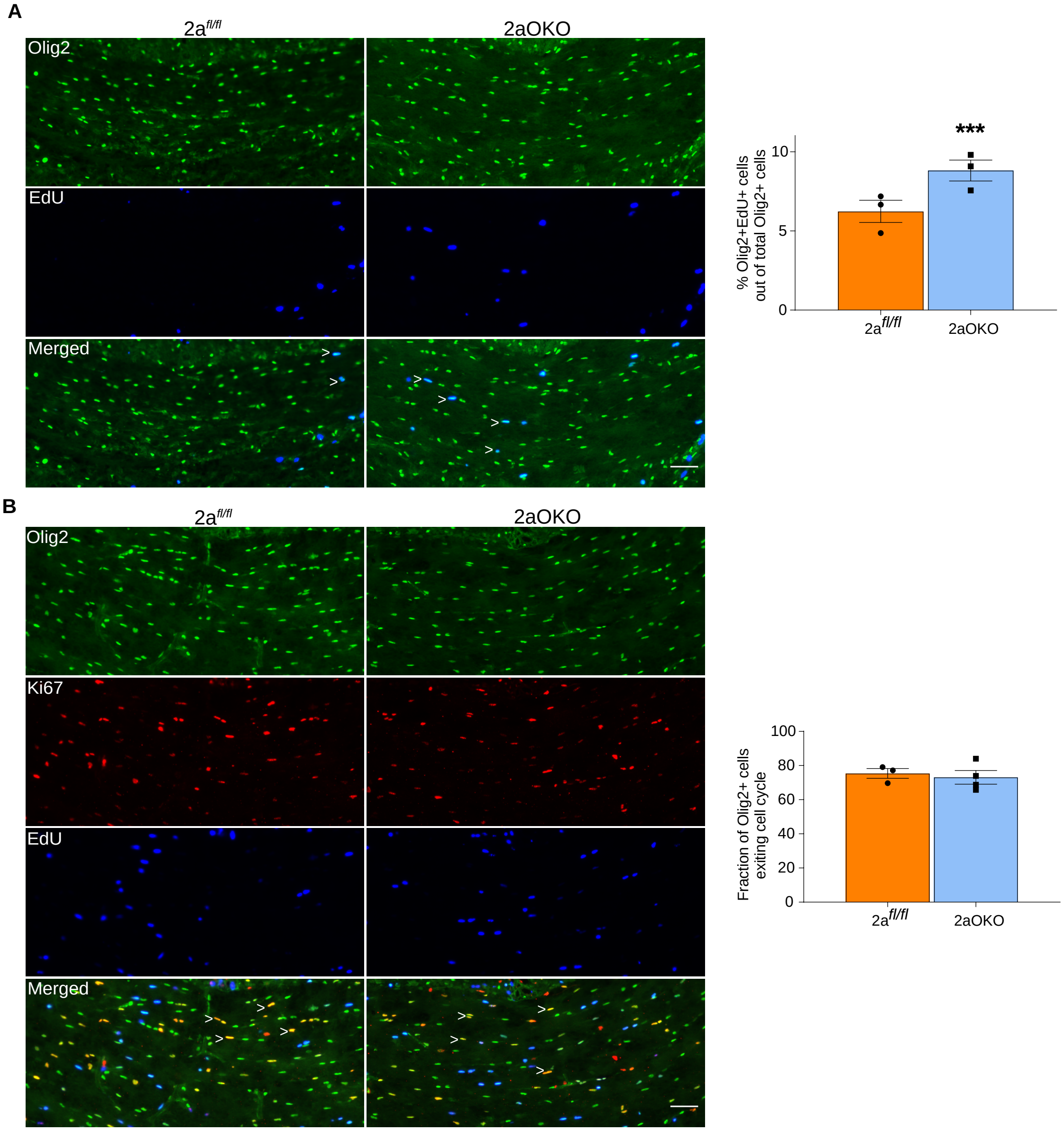

Fig S4. OPC-specific Mfsd2a deficiency affects oligodendrocyte precursor cell cycle progression. (**A**) P8 2aOKO and $2a^{fl/fl}$ controls were labelled with 250µg of EdU for 3h. IF analysis of brain coronal sections with Olig2 antibody and anti-EdU detection indicates significantly increases in percentage of proliferating Olig2+ cells (Olig2+EdU+/Olig2+) in the corpus callosum of 2aOKO relative to $2a^{fl/fl}$ controls, n = 3 per genotype. (**B**) P8 2aOKO and $2a^{fl/fl}$ controls were labelled with 250µg of EdU for 24h. IF analysis of brain coronal sections with Olig2, Ki67 antibodies and anti-EdU detection conjugate indicates no major change in fraction of Olig2+ cells undergoing cell cycle exit ((EdU+ - Ki67+ EdU+) / EdU+) in the corpus callosum of 2aOKO relative to $2a^{fl/fl}$ controls, n = 3 - 4 per genotype. Representative images for panels (**A** and **B**) are also provided. Scale bar = 50µm. Double (**A**) or triple (**B**) colocalization of Olig2+ cells with indicated antibody marker and/or EdU conjugate are identified with white arrows. Data are represented as mean ± S.E.M. ***p < 0.001 by 2-tailed Student's t-test (unpaired).

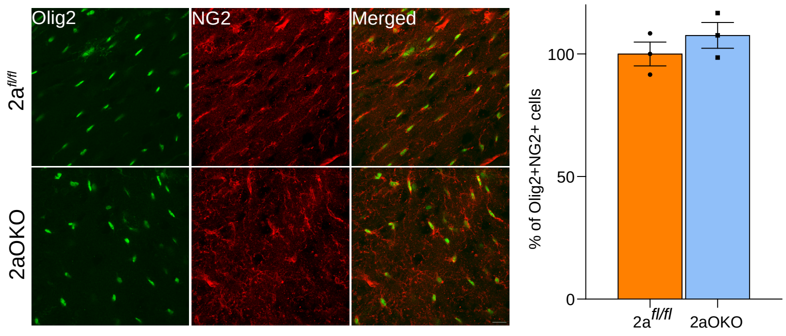

Fig S5. Mfsd2a deficiency in OPCs does not affect its total cell population numbers. Double immunostaining of P8 brain coronal sections with NG2 and Olig2 antibodies indicates similar NG2+ OPC population numbers at corpus callosum in both 2a$^{fl/fl}$ and 2aOKO, Scale bar = 20µm. Data are represented as mean ± S.E.M, n = 3 per genotype.

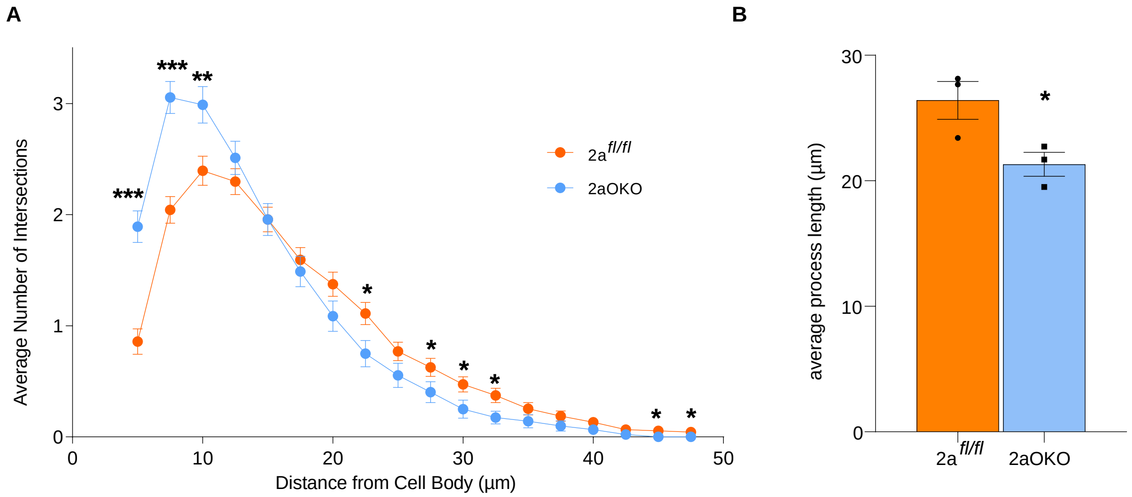

Fig S6. Mfsd2a deficiency in OPCs alters their morphology. Sholl analysis of images used in Fig 2E confirms increased numbers of branched/stunted OPCs as calculated by increased intersections of cell processes close to the cell body (**A**) and decreased average process length (**B**) in 2aOKO compared to 2a$^{fl/fl}$ controls Data are represented as mean ± S.E.M, n = 3-4 per genotype.***p < 0.0001, **p < 0.005, *p < 0.01 by 2-tailed Student's t-test (unpaired).
